# Progressive cervical cord atrophy parallels cognitive decline in Alzheimer’s disease

**DOI:** 10.1038/s41598-024-67389-9

**Published:** 2024-09-16

**Authors:** Tim M. Emmenegger, Raoul Seiler, Paul G. Unschuld, Patrick Freund, Jan Klohs, for the Alzheimer’s Disease Neuroimaging Initiative

**Affiliations:** 1 Spinal Cord Injury Center, Balgrist University Hospital, University of Zurich, Forchstrasse 340, 8008 Zurich, Switzerland; 2 Institute for Biomedical Engineering, University of Zurich and ETH Zurich, Wolfgang-Pauli-Strasse 27, 8093 Zurich, Switzerland; 3 Department of Psychiatry, University of Geneva (UniGE), 1205 Geneva, Switzerland; 4 Division of Geriatric Psychiatry, University Hospitals of Geneva (HUG), 1226 Thônex, Switzerland; 5 Zurich Neuroscience Center (ZNZ), Winterthurer Strasse 190, 8057 Zürich, Switzerland

**Keywords:** Magnetic resonance imaging, Spinal cord, Alzheimer’s disease, Atrophy, Cognitive decline, Alzheimer's disease, Cognitive ageing

## Abstract

Alzheimer’s disease (AD) is characterized by progressive episodic memory dysfunction. A prominent hallmark of AD is gradual brain atrophy. Despite extensive research on brain pathology, the understanding of spinal cord pathology in AD and its association with cognitive decline remains understudied. We analyzed serial magnetic resonance imaging (MRI) scans from the ADNI data repository to assess whether progressive cord atrophy is associated with clinical worsening. Cervical cord morphometry was measured in 45 patients and 49 cognitively normal controls (CN) at two time points over 1.5 years. Regression analysis examined associations between cord atrophy rate and cognitive worsening. Cognitive and functional activity performance declined in patients during follow-up. Compared with controls, patients showed a greater rate of decline of the anterior–posterior width of the cross-sectional cord area per month (− 0.12%, p = 0.036). Worsening in the mini-mental state examination (MMSE), clinical dementia rating (CDR), and functional assessment questionnaire (FAQ) was associated with faster rates of cord atrophy (MMSE: r = 0.320, p = 0.037; CDR: r = − 0.361, p = 0.017; FAQ: r = − 0.398, p = 0.029). Progressive cord atrophy occurs in AD patients; its rate over time being associated with cognitive and functional activity decline.

## Introduction

Alzheimer’s disease (AD) is characterized by gradually worsening episodic memory dysfunction, comorbid psychiatric symptomatology, and biomarker changes^1–3^. AD progresses over several disease stages, including an asymptomatic preclinical stage, a prodrome of mild cognitive impairment (MCI) and finally, dementia^4,5^. Neuropathology of AD suggests a multifactorial brain disorder^6^ with aggregation of β-amyloid, neurofibrillary tangles and brain atrophy as neuropathological hallmarks^7^.

Magnetic resonance imaging (MRI) studies revealed that clinically eloquent progressive brain atrophy occurs within the pre-entorhinal cortex, entorhinal cortex, hippocampi, temporal cortices, the primary associative visual regions, thalamus, amygdala, corpus callosum and primary motor cortex in AD patients^8–14^; its magnitude is associated with cognitive impairment^9,13–15^. Furthermore, the magnitude and regional distribution of brain atrophy can be used to predict cognitive decline in patients converting from MCI to AD^11,16,17^ and distinguishes AD from other types of dementia^12^. However, experimental evidence suggests that neurodegenerative processes in AD extend beyond the brain and also affect the spinal cord^18–20^. Cord pathology could therefore explain signs of motor and autonomic dysfunction that is evident in AD and has been reported to result in early institutionalization and increased mortality in AD patients^21,22^. A previous study identified that the cervical cord of AD patients is also atrophied^20^ and its extent was associated with a lower mini-mental state examination (MMSE) score. In this study, we assessed the dynamics of cervical cord atrophy over the course of 1.5 years and its relationship with cognitive and functional changes^23,24^.

## Materials and methods

### ADNI data

**Figure 1:**
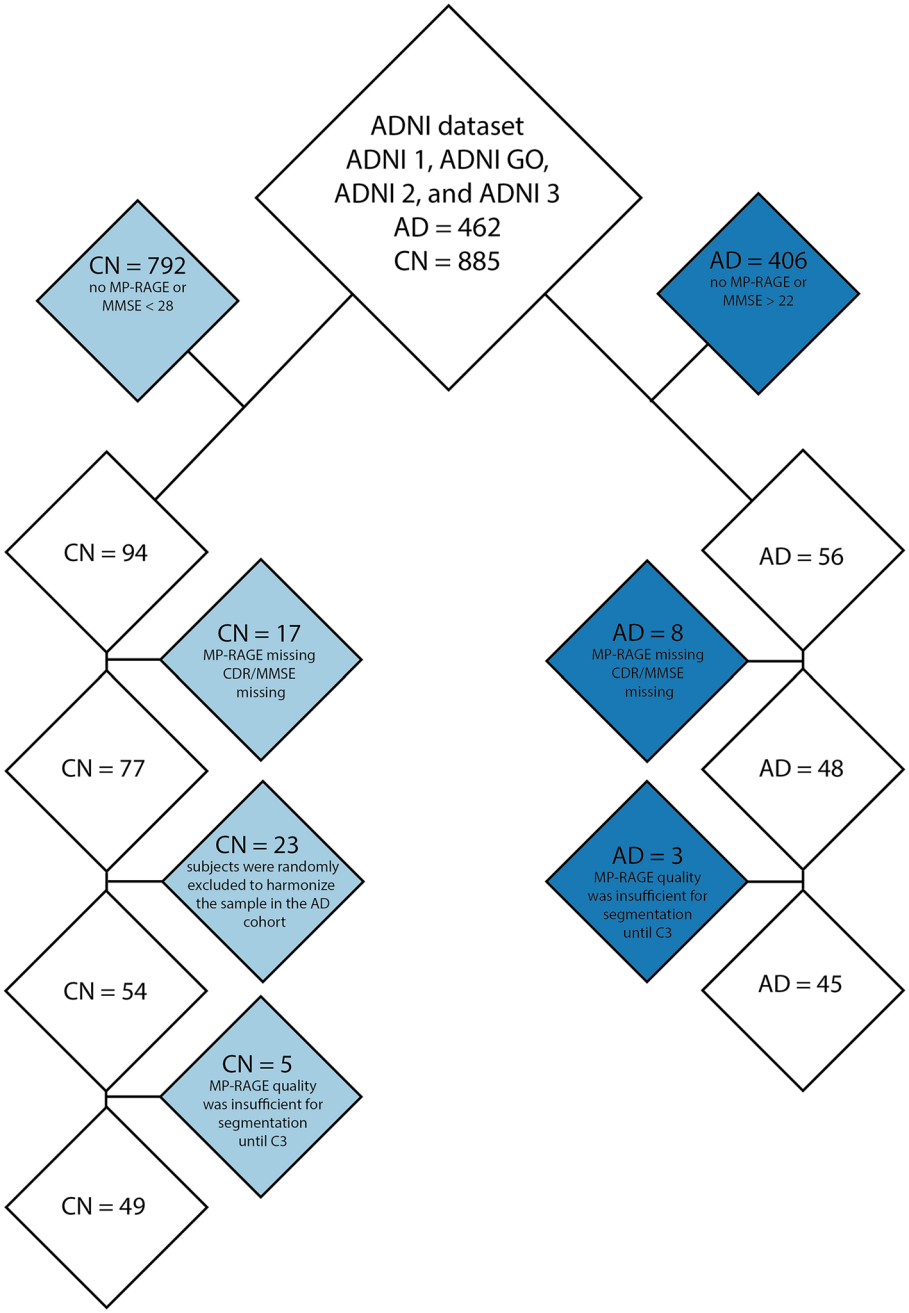
Flowchart illustrating participants’ inclusion and exclusion criteria. The flowchart depicts the process for including and excluding cognitively normal (CN, light blue) and Alzheimer's disease (AD, dark blue) patients. *MMSE* mini-mental state examination, *CDR* clinical dementia rating, *C3* third cervical level.

Data used in the preparation of this article were obtained from the Alzheimer's Disease Neuroimaging Initiative (ADNI) database (adni.loni.usc.edu). The ADNI initiative was launched in 2003 as a public & private partnership led by Principal Investigator Michael W. Weiner, MD. The primary goal of ADNI has been to test whether serial MRI, positron emission tomography (PET), other biological markers, and clinical and neuropsychological assessment can be combined to measure the progression of MCI and early AD. The ADNI investigators contributed to the design and implementation of ADNI and/or provided data but did not participate in the analysis or writing of this report. A total of 94 data sets from cognitively normal (CN) controls and AD patients were extracted, including baseline and a follow-up T1-weighted brain magnetic resonance (MR) images (latest available) that included the cervical cord, as well as MMSE and clinical dementia rating (CDR) assessments. Therefore, as of May 18th, 2022, the data search criteria for Alzheimer's disease (AD) patients were confined to the Alzheimer's Disease Neuroimaging Initiative (ADNI) datasets (ADNI 1, ADNI GO, ADNI 2, and ADNI 3) with an AD diagnosis, T1-weighted MP-RAGE images, and an MMSE score ≤ 22 at follow-up, resulting in 56 AD patients. Subsequently, AD patients with only one MP-RAGE scan or incomplete CDR or MMSE data were excluded, leaving 47 AD patients with both baseline and follow-up scans (Fig. 1). Three additional AD patients were excluded due to insufficient data quality for segmentation up to the third cervical level (Fig. 1).

For the CN group, the data search criteria, as of May 18th, 2022, were limited to the ADNI datasets (ADNI 1, ADNI GO, ADNI 2, and ADNI 3) without any diagnosis, T1-weighted MP-RAGE images, and an MMSE score ≥ 28 at the follow-up timepoint, resulting in 93 subjects. Similar to the AD group, CN subjects with only one MP-RAGE scan or incomplete CDR or MMSE data were excluded, resulting in 77 CN subjects with both baseline and follow-up scans, where 5 subjects had to excluded due to poor image quality (Fig. 1). To enhance the power in detecting disease differences, we harmonized the sample size, mean age, age range, and sex of the CN group to match the AD patients. Consequently, we randomly excluded 23 subjects to achieve this harmonization (Fig. 1).

### Image analysis

**Figure 2:**
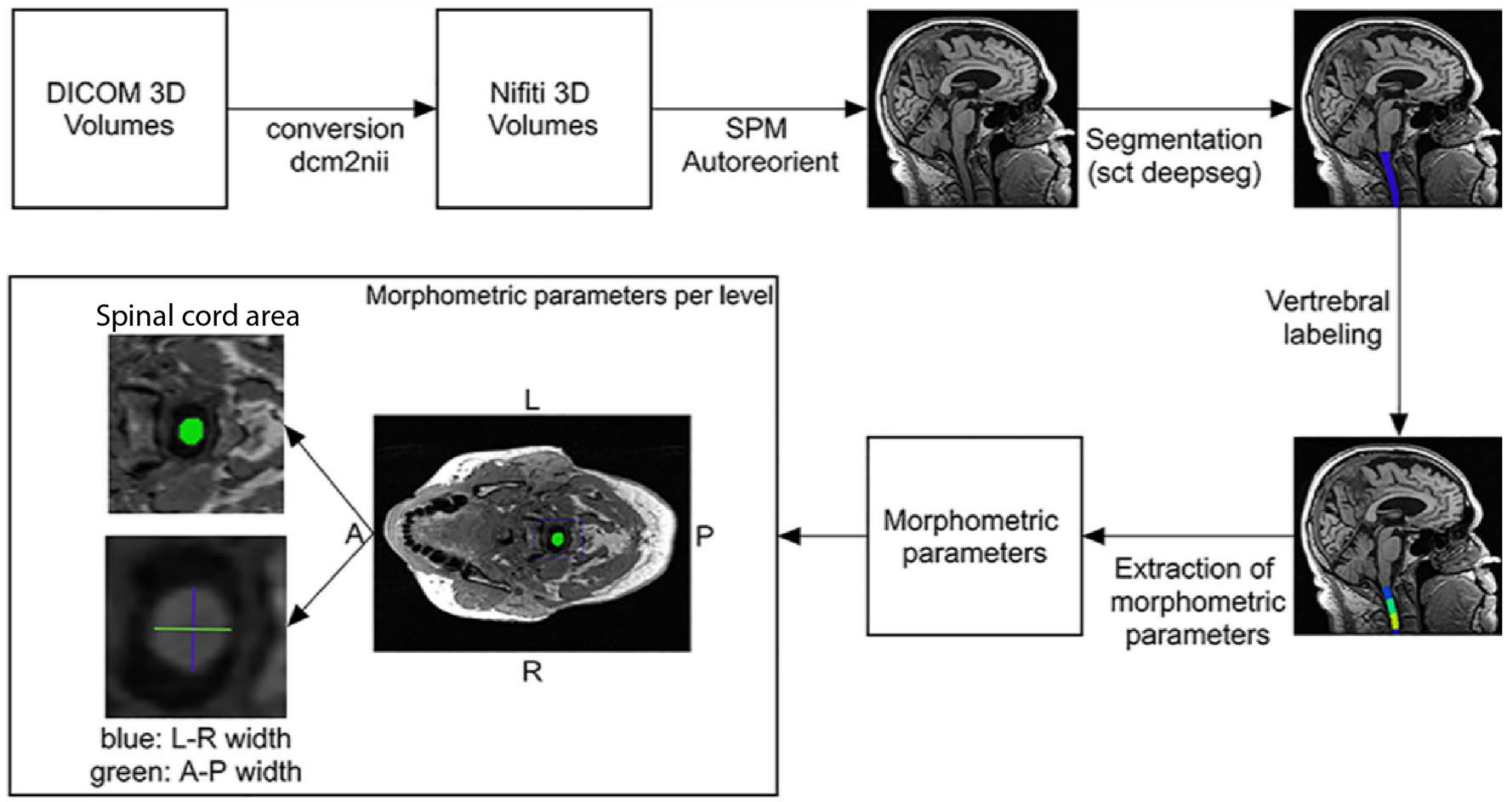
Processing pipeline to obtain morphometric parameters of the cervical spinal cord. Indicated tools were used consecutively to obtain information from T_1_-weighted magnetic resonance images of the head and neck. The spinal cord area (green area), left–right (L–R) width (green line), and anterior–posterior (A–P) width (blue line) were computed. *A* anterior; *P* posterior; *L* left; *R* right.

MRI data sets contained T_1_-weighted images acquired at 1.5 Tesla with a head radiofrequency (RF) coil and with an MP-RAGE sequence, typically 208 × 240 × 256 voxels with a voxel size of approximately 1 mm × 1 mm × 1.2 mm. The field-of-view was set to cover the cervical spinal cord up to segment C3. A total of five CN and three AD datasets had to be excluded due to poor image quality (Fig. 1). For extraction of morphometric measures of each spinal cord segment, the spinal cord was segmented and processed using Spinal Cord Toolbox (SCT) version 4.3^25^, an open-source software specifically developed to analyse spinal cord images. To minimize personal bias during the corrections, a random shuffling of the images and their segmentations was applied. The processing pipeline consisted of five steps (Fig. 2): (1) segmentation of the spinal cord using a convolutional neural network (deepseg)^26^, with a support vector machine as centerline algorithm and threshold set to 0.00015; (2) visual inspection and manual correction of the segmentations using FSL version 6.0.4^27^. Manual correction was required due to known underperformance issues of the deepseg algorithm on images acquired with head coil only^28^; (3) vertebral labelling; (4) extraction of morphometric parameters per slice; and (5) calculation of morphometric parameters per spinal cord level. Mean spinal cord area was calculated by counting pixels in each slice, which then was geometrically corrected by multiplying by the angle (in degrees) between the spinal cord centerline and inferior-superior direction. Anterior–posterior (A-P) width and left–right (L-R) width were measured by finding the major and minor axes of the spinal cord in each slice and calculating their respective length. For each vertebral level, the mean of all parameters was extracted.

Additionally, semi-automated hippocampal volumetry was carried out using a commercially available high dimensional brain mapping tool (Medtronic Surgical Navigation Technologies, Louisville, CO)^29^. Measurement of hippocampal volume is achieved first by placing manually 22 control points as local landmarks for the hippocampus on the individual brain MRI data: one landmark at the hippocampal head, one at the tail, and four per image (i.e., at the superior, inferior, medial and lateral boundaries) on five equally spaced images perpendicular to the long axis of the hippocampus. Second, fluid image transformation is used to match the individual brains to a template brain^30^. The voxels corresponding to the hippocampus are then labeled and counted to obtain volumes. This method of hippocampal volumetry has a documented reliability of an intraclass correlation coefficient better than 0.94^29^.

### Statistical analysis

The difference in sex between CN and AD groups were examined using a Fisher exact test, while the differences in age and time intervals between the two groups were assessed using a two-sample t-test. For the difference between the two groups of the MMSE and CDR change ((MMSE or CDR at follow up)—(MMSE or CDR at baseline)) a Wilcoxon rank sum test was used. To assess if the MMSE or CDR change were significantly different between baseline and follow-up within the AD group a Wilcoxon signed-rank test was used.

Statistical testing of the morphometric MRI parameters (spinal cord area, A-P, and L-R) was performed using RStudio version 1.2.1335 and R version 4.2.1.

All continuous data were tested for linearity between the grouping variables and covariate, homogeneity of regression slopes, normality using a Shapiro–Wilk test and homogeneity of variances using Levene’s test. A linear mixed model was used to estimate spinal cord area, L-R and A-P width at study inclusion (i.e. intercept analysis) and for the development between the baseline MRI scan and the follow-up. Model selection involved permutation testing of the independent parameters—age, sex, cervical level, and pathology—by evaluating model fit using the Bayesian Information Criterion (BIC). Given the longer time intervals between baseline and follow-up scan in the CN group than in the AD group, linear mixed model analysis, adjusted for the random effect of the time interval between baseline and follow-up scan, was used. Fixed effects were set as the pathology, cervical spinal cord levels, and scan time points. The random effect of follow-up scan time points, spinal cord level and pathology was also considered.

To investigate the associations between rate of decrease in clinical measures of cognition (MMSE, CDR, the Alzheimer’s Disease Assessment Scale—Cognitive (ADAS-COG)) and the functional assessment questionnaire (FAQ)^31^ with cervical cord atrophy rates, we performed a correlation test using the Pearson method, with significance set to p < 0.05. The spinal cord and disability progression changes per month were assessed by linear regression analysis, examining the relationships between rate of worsening in MMSE and CDR scores (e.g. delta MMSE/time scan rescan) and the atrophy rate averaged over C1-C3 of spinal cord area, L-R and A-P width (e.g. delta spinal cord area/time scan rescan). The same approach was also conducted using the hippocampal volume.

To investigate the association between cord morphometry and neurological and physical screening at baseline, logistic regression analysis was performed. A significance level of p < 0.05 was chosen to determine statistical significance.

### Ethical approval and informed consent

This study was approved by the local Ethics Committee and is in accordance with the Declaration of Helsinki. All participants gave informed written consent before participation.

## Results

### Patient characteristics

The patient clinical characteristics are listed in Supplementary Table 1. Data sets from 49 CN (24 females, 25 males) were selected with an MMSE score between 28 and 30 and a median age of 76 (interquartile range (IQR) 73–80). Data from 45 AD patients (22 females, 23 males) with a clinical diagnosis of AD and a total MMSE score between 17 and 27, CDR score between 2 and 10, and a median age of 76 (IQR 70–81) were selected.

The differences in the proportion of male and female subjects were not different between CN and AD patients (p = 0.996). Similarly, there were no age differences between the two groups (p = 0.137). The mean (± standard error) of the time difference between the baseline scan and follow-up MRI were significantly different between the two groups (CN: 22.53 ± 2.03 months, AD: 14.13 ± 0.91 months, p < 0.001). AD patients showed a cognitive decline in both cognitive assessments with a median MMSE decrease of − 2 (IQR − 4–0, p < 0.001) and a median CDR increase of 2 (IQR 0.5–4, p < 0.001). AD patients showed a functional decline with a median FQA increase of 5 (IQR 1–7, p < 0.001).

### Analysis of spinal cord morphometry

**Figure 3:**
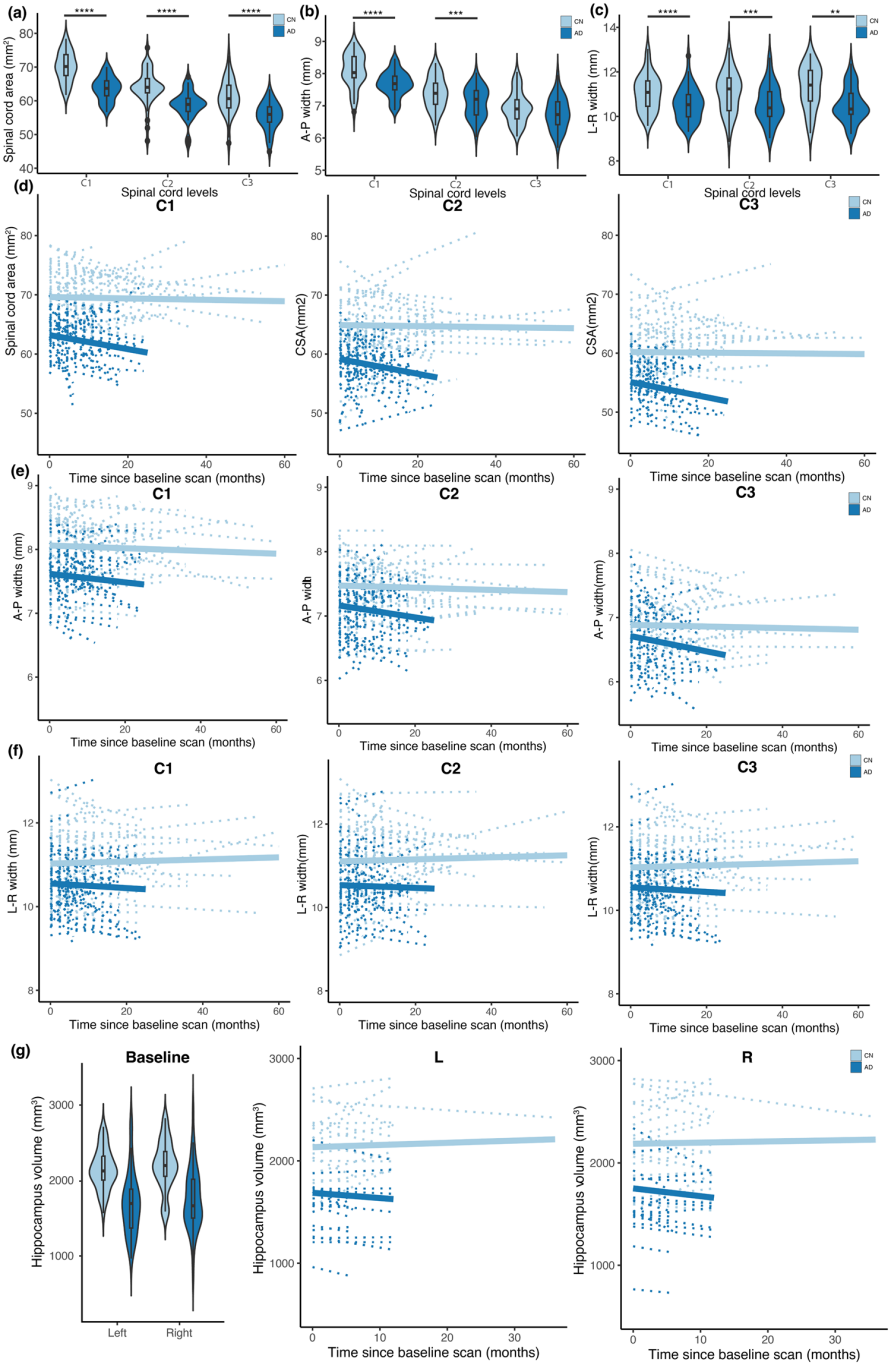
Analysis of cross-sectional and longitudinal data reveals atrophy in the cervical spinal cord in Alzheimer's disease patients. Linear mixed models with pathology, cervical spinal cord levels, and scan time points as fixed effects, and with scan time points, spinal cord level, and pathology as random effects, were used to investigate pathological differences at study inclusion (i.e., intercept analysis) and for the development between the baseline MRI scan and the follow-up. (**a**–**c**) Cross-sectional analysis of structural MRI data with spinal cord toolbox (SCT) reveals significant differences in (**a**) spinal cord area (**b**) left–right (L-R) width and (**c**) anterior–posterior (A–P) width between Alzheimer's disease (AD; dark blue) patients and cognitively normal (CN; light blue) subjects. Violin plots with overlaid boxplots showing median, 1st and 3rd quartile. *p < 0.05, **p < 0.01, ***p < 0.001, ****p < 0.0001. (**d**) Changes in spinal cord area from baseline to follow-up scan for CN and AD patients for cervical levels C1, C2 and C3. (**e**) Changes in left–right (L–R) width from baseline to follow-up scan for CN and AD patients for cervical levels C1, C2 and C3. (**f**) Changes in anterior–posterior (A–P) width from baseline to follow-up scan for CN and AD patients for cervical levels C1, C2 and C3. (**g**) Cross-sectional and longitudinal analysis of hippocampus data reveals significant differences at baseline.

At study inclusion, the spinal cord area was 5.8 mm^2^ lower in AD patients (mean 59.3 ± 0.5 mm^2^ over all levels) than it was in controls (mean over all levels 65.1 ± 0.5 mm^2^; p < 0.001; Fig. 3a and Table 1). Patients had a trend toward a significantly greater rate of change of spinal cord cross-sectional area than did controls (patients decreased by 0.18% per month more than controls, p = 0.069; Fig. 3d and Table 1). In patients only, mean spinal cord area decreased by 0.20 ± 0.12% per months (p = 0.045), whereas in controls the spinal cord area did not change substantially (− 0.02 ± 0.04% per month, p = 0.735; Fig. 3d and Table 1).

**Table 1:** Cervical cord morphometry.

MRI parameter	Estimated mean per level				AD—CN		
	CN	SE	AD	SE	Differences		P value
CSA [mm^2^]						%	
C1	69.6	0.5	63.2	0.6	− 6.4	− 9.2	< 0.001
C2	64.9	0.5	59.2	0.5	− 5.7	− 8.8	< 0.001
C3	60.2	0.7	55.1	0.7	− 5.1	− 8.5	< 0.001
A–P width [mm]							
C1	8.06	0.06	7.62	0.06	− 0.44	− 5.46	< 0.001
C2	7.47	0.06	7.16	0.06	− 0.31	− 4.15	< 0.001
C3	6.89	0.08	6.71	0.08	− 0.18	− 2.61	0.062
L–R width [mm]							
C1	11.0	0.1	10.6	0.1	− 0.4	− 3.6	0.002
C2	11.1	0.1	10.5	0.1	− 0.6	− 5.4	< 0.001
C3	11.2	0.1	10.5	0.2	− 0.7	− 6.2	0.001
Hippocampus [mm^3^]							
L	2134	43.6	1688	63.4	− 446	20.9%	< 0.001
R	2187	53.8	1751	71.0	− 436	19.9%	< 0.001

Analysis of cross-sectional structural MRI data with spinal cord toolbox (SCT) shows significant differences in spinal cord area, left–right (L-R) width, and anterior–posterior (A-P) width between Alzheimer's disease (AD) patients and cognitively normal (CN) subjects.

At study inclusion, the A-P width was 0.316 mm lower in AD patients (mean 7.18 ± 0.06 mm over all levels) than it was in controls (mean over all levels 7.50 ± 0.06 mm; p < 0.001; Fig. 3b and Table 1). Patients had a significantly greater rate of change of A-P width than did controls (patients decreased by 0.12% per month more than controls, p = 0.036; Fig. 3b and Table 1). In patients mean A-P width decreased by 0.13 ± 0.04% per month (p = 0.005) whereas in controls the A-P width did not change substantially (− 0.01 ± 0.03% per month, p = 0.449; Fig. 3e and Table 1).

At study inclusion, the L-R width was 0.566 mm lower in AD patients (mean 10.5 ± 0.1 mm over all levels) than it was in controls (mean over all levels 11.1 ± 0.1 mm, p < 0.001; Fig. 3c and Table 1). Patients had a similar rate of change of L-R width as controls (patients decreased by 0.01% per month more than controls, p = 0.319; Fig. 3c and Table 1). In patients mean L-R width did not change substantially (AD = − 0.03 ± 0.1% per month, p = 0.385) as in controls (CN = 0.02 ± 0.04% per month, p = 0.552; Fig. 3f and Table 1).

### Analysis hippocampal volume

At study inclusion, the hippocampal volume was 441 ± 79.9 mm^3^ lower in AD patients (mean left and right: 1719 ± 65.2 mm^3^) than it was in controls (mean left and right: 2160 ± 46.1 mm^3^; p < 0.001; Fig. 3g and Table 1). Patients had a trend significance towards a greater rate of change of hippocampal volume than did controls (patients decreased by 0.47% per month more than controls, p = 0.051; Fig. 3g and Table 1). In patients, mean hippocampal volume decreased by 0.37 ± 0.20% per month (p = 0.041) whereas in controls the hippocampal volume did not change substantially (0.08 ± 0.16% per month p = 0.311, Fig. 3g and Table 1).

### Associations between pathology and cognitive and functional impairment

**Figure 4:**
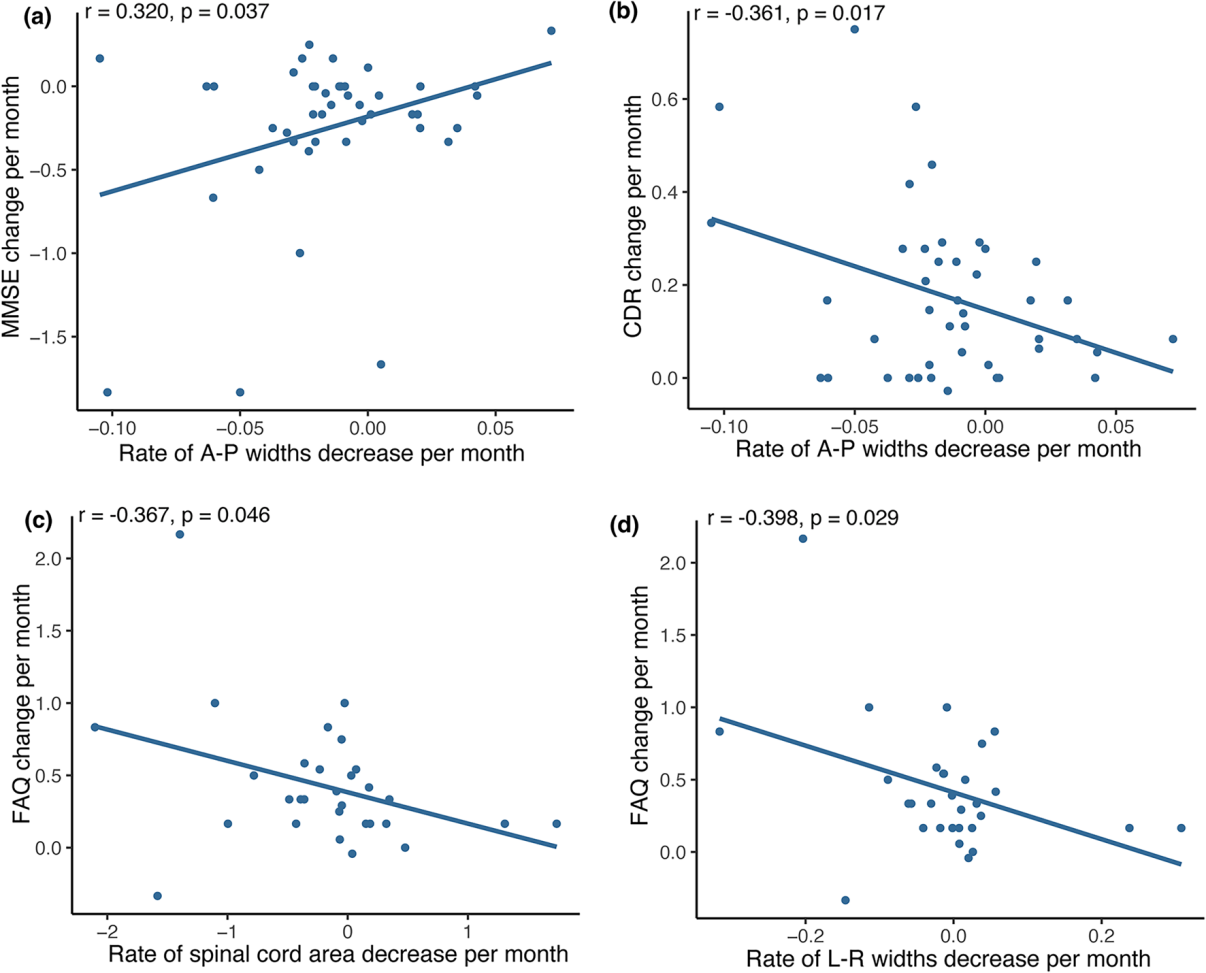
Linear regression of morphological parameters with clinical measures of cognitive impairment. Linear regression analysis, examining the relationships between rate of worsening in MMSE (Mini-Mental State Examination), CDR (Clinical Dementia Rating), and FAQ (Functional Assessment Questionnaire) scores (e.g., delta MMSE/time scan rescan) and the atrophy rate averaged over C1–C3 of spinal cord area, L–R and A–P width (e.g., delta spinal cord area/time scan rescan), using Pearson correlation. (**A**) Correlation of anterior–posterior (A-P) width change per month for the cervical spinal cord level C1–C3 of Alzheimer's disease (AD) patients with MMSE change per month and (**b**) CDR change per month. Correlation of (**c**) spinal cord area and (**d**) left–right (L-R) width change per month for the cervical spinal cord level C1-C3 of Alzheimer's disease (AD) patients with FAQ change per month. Dark blue regression line with dark blue circle for individual subject values.

At baseline, no significant association between cord morphometry and neurological and physical screening was observed in patients. However, an association between the cognitive decline per month (i.e., MMSE decline per month, CDR increase per month) and A-P width decrease per month was observed (MMSE: r = 0.320, p = 0.037; CDR: r = − 0.361, p = 0.017; Fig. 4). The association between the cognitive decline per month and the hippocampal volume decrease per month was not significant (MMSE: r = − 0.397, p = 0.115; CDR: r = 0.373, p = 0.140). No significant association was found between ADAS-COG change and hippocampal volume (p > 0.360) nor were any significant cord changes observed (p > 0.075). The spinal cord area and the L-R width decrease per month were significantly associated with the FAQ change (spinal cord area: p = 0.046, r = − 0.367; L-R width: p = 0.029, r = − 0.398), but the hippocampal volume was not correlated with the FAQ change (p > 0.853).

## Discussion

Brain atrophy is a neuropathological hallmark of AD that has been corroborated in numerous MRI neuroimaging studies^8–14^. Using longitudinal data generated by ADNI^32^, we found that the already atrophied cervical cord, further progressed during the 1.5-year follow-up when compared to CN. This finding is consistent with an earlier cross-sectional study on cervical pathology in AD^20^. The magnitude of spinal cord area atrophy in comparison to the CN ranged from 10.26 to 11.76% at follow-up, which is in line with the reported values^20^ with the order of 10%. In addition to the cross-sectional area, we also assessed the L-R and A-P widths, which are proxies for lateral tract and dorsal column atrophy^33–36^. All three measures (spinal cord area, L–R and A–P widths) were decreased across segments C1–C3 in AD patients compared to CN at baseline and follow-up. Studies in transgenic models of AD revealed axonal degeneration and defective axonal transport along with spinal cord neuropathology^37–41^, as well as a progressive gradient of p-tau deposits in humans along the spinal cord with increased p-tau deposition closer to the brain^19^. We evaluated the possibility of a progressive character in cervical cord atrophy by analyzing longitudinal structural MRI data. Atrophy rates in cervical spinal cord segments were similar to what has been observed in brain regions in AD patients^42^. The observed cervical cord changes were mainly driven by changes in the A-P width. Changes in the A-P width have been associated with dorsal column damage in patients suffering from spinal cord injury^33–36^. Hence, these changes might be a marker of proprioceptive dysfunction in AD that would explain increased falls^43^ and also acceleration of cognitive decline due to loss of sensory input^44^.

The presence of cord atrophy at the initial time point of the study suggests that neurodegeneration is already advanced at this stage of the disease^21,45,46^. Moreover, it is important to consider that in the present study, AD patients were part of a senior population with a median age of 76 years. It has been observed that older AD patients exhibit reduced rates of cortical atrophy compared to younger AD patients, likely due to the more advanced stage of neurodegeneration in the older population^47^. Based on these supraspinal findings, it may be likely that cervical cord atrophy rates are higher in AD at a younger age or at earlier disease stages, which needs to be tested in further studies. Thus, to understand the dynamics of spinal cord pathology, future studies are warranted that include patients with preclinical AD or with MCI and including more patients and time points and MRI with sufficient GM and WM contrast.

We found associations between monthly cord morphometry and cognitive decline rates, while hippocampal volume changes showed no significant association with cognitive decline. Specifically, AD patients with less cord pathology showed less MMSE score decreases, and less CDR score increases until the follow-up timepoint. In previous publications, it has been shown that cognitive function is related to the integrity of cortical structures and the hippocampus^9,13–15^. However, in this cohort, we were not able to confirm these findings, which could be due to the known decrease in atrophy rates in later stages of AD, which might be due to the advanced stage of the disease. Similarly, confirming this finding, publications with similar patients age and cognitive impairments were not able to provide a significant association with cognitive decline and atrophy rates of any cranial structures^13^. Furthermore there is clear evidence that the correlation is less prominent in individuals with extensive atrophy at baseline^14^. The spinal cord, however, is not directly related to cognition, particularly memory. Nevertheless, since the spinal cord relays sensory, motor, and autonomous function between the brain and periphery of the body, cervical cord atrophy may also impact to some extent these commonly used clinical test scores. For example, some of the cognitive tasks assessed in the MMSE scores depend on motor and sensory function^23^. The involvement of the cervical cord in AD pathology highlights that motor, sensory, and autonomic dysfunction should also be assessed in the clinical examination of AD patients^48–52^ particularly as it seems that the decrease in the hippocampus was not associated with the MSSE and CDR anymore in our cohort. The FAQ change, however, which is more closely related to motor function, was found to be significantly correlated with the decline in spinal cord area and its L-R width. Particularly, the L-R width has been demonstrated to be a valuable proxy for the corticospinal tract and has consistently been shown to predict motor function recovery in spinal cord injury patients^33–36^.

This study has some limitations. It retrospectively investigates spinal cord morphometry and its association with clinical assessments of cognitive and functional decline. Therefore, direct assessments of functional deterioration, such as those pertaining to motor or sensory function, were not performed. However, a significant association was observed with cognitive decline and the functional assessment questionnaire, while no significant association was found with hippocampal volume decreases and cognitive decline and the FAQ. In this study, only a limited age range (IQR 70–81), and cognitive impairment (MMSE score from 17 to 27, CDR score from 2 to 10) were chosen to be comparable with previous publications, limiting the power to investigate whether spinal cord morphometry could serve as a neuroimaging marker in early disease stages, or potential age-dependent interactions. Nevertheless, this study lays the foundation for understanding how spinal cord morphometry changes in the later stages of AD and how these changes are associated with some crucial assessments in AD.

In conclusion, progressive cervical cord atrophy is clinically eloquent in AD, as it is associated with cognitive decline. Future MRI studies are justified to enhance our understanding of neuropathological processes and explore potential opportunities for therapeutic intervention in the spinal cord of AD patients.

## Supplementary Information

Supplementary Information 1.

Supplementary Table 1.

## Data Availability

The data that support the findings of this study are available from the corresponding author upon reasonable request. All ADNI data are deposited in a publicly accessible repository and can be accessed at adni.loni.usc.edu.

## Abbreviations

AD: Alzheimer's disease
ADNI: Alzheimer's Disease Neuroimaging Initiative
A-P: Anterior–posterior
CDR: Clinical dementia rating
CN: Cognitive normal
IQR: Interquartile range
L-R: Left–right
MCI: Mild cognitive impairment
MMSE: Minimal-mental state examination
MR: Magnetic resonance
MRI: Magnetic resonance imaging
PET: Positron emission tomography
RF: Radiofrequency
SCT: Spinal cord toolbox
