# Supplementary material for: Progressive cervical cord atrophy parallels cognitive decline in Alzheimer’s disease
Source: Sci Rep. 2024 Sep 16;14:21595. doi: 10.1038/s41598-024-67389-9 (PMC11405669; doi:10.1038/s41598-024-67389-9)
Supplement: Supplementary file 1 — Supplementary Information 1. [file 41598_2024_67389_MOESM1_ESM.pdf]

Original

**Spinal cord Area**							
		Estimate	Std.Error	df	t	value	Pr(>|t|)
	(Intercept)	69.625417	0.527323	125.456577	132.036	<2.00E-16	***
	patient	-6.42782	0.766398	128.205751	-8.387	7.76E-14	***
	levels	-4.709506	0.325762	139.858449	-14.457	<2.00E-16	***
	timepoint	-0.011814	0.029068	42.094276	-0.406	0.686	
	levels:timepoint	0.002847	0.013844	260.68414	0.206	0.837	
	patient:timepoint	-0.106571	0.08016	41.232781	-1.329	0.191	
	patient:levels	0.66487	0.471887	143.327912	1.409	0.161	
	patient:levels:timepoint	-0.009701	0.029155	245.572729	-0.333	0.74	

	contrast			estimate	SE	df	t.ratio	p.value
baseline differences	patient0	-	patient1	5.79	0.755	113	7.677	<.0001
Atrophy rates differences	patient0	-	patient1	0.116	0.0769	60.2	1.506	0.0687
	patient	timepoint.tre	SE	df	t.ratio	p.value		
	0	-0.00909	0.0267	45.5	-0.341	0.735		
	1	-0.12493	0.0721	47.5	-1.732	0.0449		

**LR-width**							
		Estimate	Std. Error	df	t	value	Pr(>|t|)
	(Intercept)	1.10E+01	1.08E-01	1.03E+02	102.588	<2.00E-16	***
	patient	-4.75E-01	1.56E-01	1.04E+02	-3.053	0.00288	**
	levels	7.86E-02	5.63E-02	1.24E+02	1.394	0.16568	
	timepoint	2.47E-03	4.31E-03	3.77E+01	0.573	0.56977	
	levels:timepoint	-5.25E-05	1.86E-03	2.43E+02	-0.028	0.97757	
	patient:timepoint	-8.01E-03	1.27E-02	4.35E+01	-0.629	0.53254	
	patient:levels	-9.45E-02	8.14E-02	1.25E+02	-1.161	0.24786	
	patient:levels:timepoint	2.25E-03	3.89E-03	2.30E+02	0.578	0.56397	

	contrast			estimate	SE	df	t.ratio	p.value
baseline differences	patient0	-	patient1	0.566	0.164	119	3.451	0.0004
Atrophy rates differences	patient0	-	patient1	0.00586	0.0124	57.5	0.472	0.3193

	patient	timepoint.tre	SE	df	t.ratio	p.value		
	0	0.00242	0.00404	46.2	0.599	0.5522		
	1	-0.00344	0.01174	46.7	-0.293	0.3853		

**AP-width**							
		Estimate	Std. Error	df	t	value	Pr(>|t|)
	(Intercept)	8.06E+00	5.87E-02	1.29E+02	137.435	<2.00E-16	***
	patient	-4.44E-01	8.52E-02	1.32E+02	-5.218	6.85E-07	***
	levels	-5.88E-01	3.62E-02	1.53E+02	-16.253	<2.00E-16	***
	timepoint	-2.14E-03	2.58E-03	3.85E+01	-0.829	0.412	
	levels:timepoint	4.36E-04	1.50E-03	2.97E+02	0.291	0.771	
	patient:timepoint	-4.51E-03	4.90E-03	1.12E+02	-0.92	0.36	
	patient:levels	1.35E-01	5.24E-02	1.56E+02	2.573	0.011	*
	patient:levels:timepoint	-3.05E-03	3.14E-03	2.80E+02	-0.974	0.331	

	contrast			estimate	SE	df	t.ratio	p.value
baseline differences	patient0	-	patient1	0.316	0.0849	119	3.717	0.0002
Atrophy rates differences	patient0	-	patient1	0.00743	0.00406	71	1.83	0.0357
	patient	timepoint.tre	SE	df	t.ratio	p.value		
	0	-0.00172	0.00225	41.6	-0.764	0.4494		
	1	-0.00915	0.00338	40.9	-2.709	0.0049		

Correlation	rateMMSE		rateCDR		rateADAS-Cog		rateFAQ	
	r	p-value	r	p-value	r	p-value	r	p-value
rateCSA	0.059	0.059	0.252	0.252	0.075	0.075	0.046	0.046
rateAP	0.037	0.037	0.017	0.017	0.114	0.114	0.386	0.386
rateLR	0.179	0.179	0.947	0.947	0.169	0.169	0.029	0.029

With age and sex

**Spinal cord Area**							
		Estimate	Std.Error	df	t	value	Pr(>|t|)
	(Intercept)	7.02E+01	4.06E+00	9.51E+01	17.275	<2.00E-16	***
	patient	-6.42E+00	7.63E-01	1.28E+02	-8.405	7.15E-14	***
	levels	-4.71E+00	3.27E-01	1.38E+02	-14.383	<2.00E-16	***
	timepoint	-1.18E-02	2.96E-02	4.35E+01	-0.398	0.692	
	age	5.82E-04	5.24E-02	9.44E+01	0.011	0.991	
	gender	-1.28E+00	6.93E-01	9.26E+01	-1.847	0.068	.
	levels:timepoint	2.20E-03	1.38E-02	2.64E+02	0.159	0.874	
	patient:timepoint	-1.07E-01	8.07E-02	4.17E+01	-1.326	0.192	

	patient:levels	6.60E-01	4.74E-01	1.42E+02	1.393	0.166	
	patient:levels:timepoint	-8.77E-03	2.90E-02	2.49E+02	-0.302	0.763	

	contrast			estimate	SE	df	t.ratio	p.value
baseline differences	patient0	-	patient1	5.78	0.761	116	7.597	<.0001
Atrophy rates differences	patient0	-	patient1	0.115	0.0776	60.7	1.485	0.0713
	patient	timepoint.tre	SE	df	t.ratio	p.value		
	0	-0.00969	0.0273	48.1	-0.354	0.7247		
	1	-0.12501	0.0729	48.2	-1.715	0.0464		

**LR-width**							
		Estimate	Std. Error	df	t	value	Pr(>|t|)
	(Intercept)	1.03E+01	8.56E-01	9.43E+01	12.08	<2.00E-16	***
	patient	-4.50E-01	1.53E-01	1.05E+02	-2.942	0.00402	**
	levels	7.93E-02	5.66E-02	1.23E+02	1.401	0.16362	
	timepoint	1.61E-03	4.42E-03	4.11E+01	0.365	0.71696	
	age	1.11E-02	1.11E-02	9.41E+01	1.006	0.31715	
	gender	-3.40E-01	1.47E-01	9.34E+01	-2.315	0.0228	*
	levels:timepoint	-1.73E-04	1.86E-03	2.47E+02	-0.093	0.92597	
	patient:timepoint	-7.95E-03	1.28E-02	4.39E+01	-0.622	0.53719	
	patient:levels	-9.57E-02	8.18E-02	1.24E+02	-1.17	0.24413	
	patient:levels:timepoint	2.44E-03	3.87E-03	2.35E+02	0.63	0.52933	

	contrast			estimate	SE	df	t.ratio	p.value
baseline differences	patient0	-	patient1	0.541	0.163	123	3.321	0.0006
Atrophy rates differences	patient0	-	patient1	0.00562	0.0125	57.6	0.45	0.3271
	patient	timepoint.tre	SE	df	t.ratio	p.value		
	0	0.00145	0.00418	51.4	0.347	0.7302		
	1	-0.00417	0.01183	47.2	-0.353	0.3629		

**AP-width**							
		Estimate	Std. Error	df	t	value	Pr(>|t|)
	(Intercept)	8.53E+00	4.51E-01	9.45E+01	18.89	<2.00E-16	***
	patient	-4.59E-01	8.55E-02	1.32E+02	-5.371	3.42E-07	***
	levels	-5.88E-01	3.61E-02	1.52E+02	-16.312	<2.00E-16	***
	timepoint	-1.65E-03	2.62E-03	4.07E+01	-0.63	0.5319	
	gender	4.72E-02	7.75E-02	9.27E+01	0.61	0.5435	

	age	-6.37E-03	5.82E-03	9.37E+01	-1.095	0.2764	
	levels:timepoint	4.62E-04	1.50E-03	2.98E+02	0.309	0.7578	
	patient:timepoint	-4.48E-03	4.92E-03	1.11E+02	-0.91	0.3648	
	patient:levels	1.35E-01	5.22E-02	1.55E+02	2.588	0.0106	*
	patient:levels:timepoint	-3.12E-03	3.14E-03	2.81E+02	-0.994	0.3209	

	contrast			estimate	SE	df	t.ratio	p.value
baseline differences	patient0	-	patient1	0.33	0.086	122	3.839	0.0001
Atrophy rates differences	patient0	-	patient1	0.00746	0.00408	71.2	1.827	0.0359
	patient	timepoint.tre	SE	df	t.ratio	p.value		
	0	-0.00121	0.00231	45.5	-0.524	0.6025		
	1	-0.00867	0.00343	42.6	-2.53	0.0076		

Correlation	rateMMSE		rateCDR		rateADAS-Cog		rateFAQ	
	r	p-value	r	p-value	r	p-value	r	p-value
rateCSA	0.2381978	0.056	-0.039	0.381	-0.380	0.0482	-0.251	0.011
rateAP	4.835	0.029	-1.563	0.042	-6.281	0.102	-3.048839	0.1292
rateLR	1.2262839	0.183	0.019	0.953	-2.170	0.103	-1.689	0.012
