# Supplementary material for: Progressive cervical cord atrophy parallels cognitive decline in Alzheimer’s disease
Source: Sci Rep. 2024 Sep 16;14:21595. doi: 10.1038/s41598-024-67389-9 (PMC11405669; doi:10.1038/s41598-024-67389-9)
Supplement: Supplementary file 2 — Supplementary Table 1. [file 41598_2024_67389_MOESM2_ESM.docx]

**Table 1.** Patient clinical characteristics.

|  | CN  (n = 49) | AD  (n = 45) |
| --- | --- | --- |
| Demographic data |  |  |
| Age, median (IQR) | 77 (75-82) | 77 (70-82) |
| Female, n (%) | 24 (49%) | 21 (47%) |
| CDR, median (IQR) | 0 (0-0) | 5 (4-6) |
| MMSE, median (IQR) | 30 (30-30) | 19 (17-22) |
| APOE ε4 genotype, n (%) |  |  |
| APOE ε4 0 | 36 (73%) | 15 (33%) |
| APOE ε4 1 | 12 (25%) | 18 (40%) |
| APOE ε4 genotype | 1 (2%) | 12 (27%) |
| Risk factors |  |  |
| Cardiovascular diseases (%) | 34 (69%) | 28 (62%) |
| Endocrine-metabolic disease (%) | 14 (29%) | 16 (36%) |
| Alcohol consumption (%) | 1 (2%) | 2 (4%) |
| Smoking (%) | 14 (17%) | 17 (38%) |

Numbers (n) and percentage (%) or median and interquartile range (IQR) are shown as also numbers of Apolipoprotein E (APOE) ε4 alleles. CDR = clinical dementia rating, MMSE = minimal-mental state examination.
